# The relation between inspiratory muscle strength and bacterial colonization and other clinical factors in patients with non-cystic fibrosis bronchiectasis

**DOI:** 10.5578/tt.20239914

**Published:** 2023-06-13

**Authors:** İ. CANDEMİR, P. ERGÜN, N. DEMİR, F. TAŞDEMİR

**Affiliations:** 1 Clinic of Chest Disease, Atatürk Sanatoryum Education and Research Hospital, Ankara, Türkiye

**Keywords:** Bronchiectasis, respiratory muscle, infections

## Abstract

**ABSTRACT:**

The relation between inspiratory muscle strength and bacterial colonization and other clinical factors in patients with non-cystic fibrosis bronchiectasis

**Introduction:**

This study aimed to investigate whether inspiratory muscle strength was associated with bacterial colonization and other clinical outcomes and whether bacterial colonization was associated with clinical outcomes in patients with non-cystic fibrosis bronchiectasis (NCFB)

**Materials and Methods:**

Eighty-six patients were enrolled in a cross-sectional study. Patients were divided into two groups according to the presence of inspiratory muscle weakness and bacterial colonization. Parameters were compared between groups.

**Results:**

Bronchiectasis etiologies were post-infectious, Kartagener’s syndrome, and primary ciliary dyskinesia. The median value of MIP was -68, and MEP was 89 cm H_2_O in all patients. Although the ratio of bacterial colonization was similar to patients without inspiratory muscle weakness, the inspiratory muscle weakness group had a higher number of females, lower FEV_1_, FVC, ISWT, CRQ, higher MRC, E-FACED, SGRQ, number of hospitalization (p< 0.05). When colonized and non-colonized patients were compared, MIP, and MEP were similar in spite of adjusted BMI, age, and sex. FEV_1_, FVC, ISWT, and ESWT were lower, and E-FACED scores (p< 0.05) were higher in colonized patients

**Conclusion:**

Although inspiratory muscle strength was not associated with bacterial colonization in NCFB patients, it is an important factor that could be linked to disease severity, pulmonary functions, quality of life, and exercise capacity. Bacterial colonization was also associated with severe disease, deteriorated pulmonary functions, and exercise capacity.

## Abstract (tr)

**ÖZ:**

Kistik fibrozis dışı bronşektazili hastalarda inspiratuvar kas gücü ile bakteri kolonizasyonu ve diğer klinik faktörler arasındaki ilişki

**Giriş:**

Bu çalışmanın amacı, kistik fibrozis dışı bronşektazili (NCFB) hastalarda inspiratuvar kas kuvvetinin bakteriyel kolonizasyonla, diğer klinik sonuçlarla ve bakteriyel kolonizasyonun klinik sonuçlarla ilişkili olup olmadığını araştırmaktır.

**Materyal ve Metod:**

Seksen altı hasta kesitsel çalışmaya alındı. Hastalar inspiratuvar kas güçsüzlüğü ve bakteri kolonizasyonu varlığına göre iki gruba ayrıldı. Parametreler gruplar arasında karşılaştırıldı.

**Bulgular:**

Bronşektazi etiyolojileri postenfeksiyöz, Kartagener sendromu, primer siliyer diskineziydi. Tüm hastalarda ortanca MIP değeri -68, MEP 89 cm H_2_O idi. Bakteriyel kolonizasyon oranı inspiratuvar kas güçsüzlüğü olmayanlara benzer olmasına rağmen, inspiratuvar kas güçsüzlüğü grubunda kadın sayısı daha fazla, daha düşük FEV_1_, FVC, ISWT, CRQ ve MRC, E-FACED, SGRQ, hastanede yatış sayısı daha yüksek bulundu (p< 0,05). Kolonize olan ve kolonize olmayan hastalar karşılaştırıldığında, MIP, MEP değerleri düzeltilmiş BMI, yaş, cinsiyete rağmen benzerdi. Kolonize hastalarda FEV_1_, FVC, ISWT, ESWT daha düşük, E-FACED skorları (p< 0,05) daha yüksekti

**Sonuç:**

NCFB hastalarında inspiratuvar kas kuvveti bakteri kolonizasyonu ile ilişkili olmasa da hastalık şiddeti, solunum fonksiyonları, yaşam kalitesi, egzersiz kapasitesiyle ilişkilendirilebilecek önemli bir faktördür. Bakteriyel kolonizasyon ayrıca ağır hastalık, kötüleşen akciğer fonksiyonları ve egzersiz kapasitesi ile ilişkiliydi.

## Introduction

Bronchiectasis is a chronic disease characterized by persistent and abnormal anatomic dilatation and thickening of the bronchi with an inflammatory response in the lumen that results in reduced mucociliary clearance and recurrent lung infections ( 1 ). There are many causes of non-cystic fibrosis bronchiectasis (NCFB) ( 2 , 3 ). Major symptoms are cough, excessive secretions, dyspnea, exercise intolerance, and fatigue ( 1 ). Respiratory muscle weakness, in patients with bronchiectasis, has been shown in a few studies ( 4 , 5 ). Although the main mechanism of respiratory muscle weakness has not been revealed, possible mechanisms may include primary weakness or hyperinflation-related functional weakness ( 6 ). As a result of respiratory muscle weakness, cough, and airway clearance are insufficient. Without effective mucociliary and innate antimicrobial defenses, there is a higher risk of chronic bacterial colonization of the airways contributing to airway inflammation and structural damage, which is known as the vicious cycle hypothesis ( 7 ).

Airway bacterial colonization plays an important role in the pathogenesis and prognosis of bronchiectasis. Bacterial colonization has been found to be linked with disease severity. Additionally, in recent studies, it has been demonstrated that patients with bronchiectasis with airway bacterial colonization have a greater risk of exacerbation, hospitalization, and mortality ( 8 , 9 ).

There are several studies about bronchiectasis and colonization; however, few studies have investigated the relation between respiratory muscle strength and bacterial colonization, and other clinical parameters. The primary aim of this study was to investigate whether inspiratory muscle strength was associated with bacterial colonization and other clinical outcomes in patients with NCFB, and the secondary aim was to establish whether bacterial colonization was associated with other clinical outcomes in these patients.

## MATERIALS and METHODS

### Study Design

This was a cross-sectional study. The data were obtained from the database of our pulmonary rehabilitation (PR) center regarding patients who had been evaluated from January 2013 to December 2018. Informed consent containing information about PR was obtained before performing PR. In the consent form, it was mentioned that the evaluated parameters and information of the patients would be recorded. Approval for the study was obtained from the institutional review board.

### Patient Characteristics

The diagnoses of the patients were confirmed by the same chest physician according to the history, physical examination, chest X-ray, and high-resolution computed tomography (HRCT) of the thorax.

### The Inclusion Criteria Were As Follows

**Figure 1:**
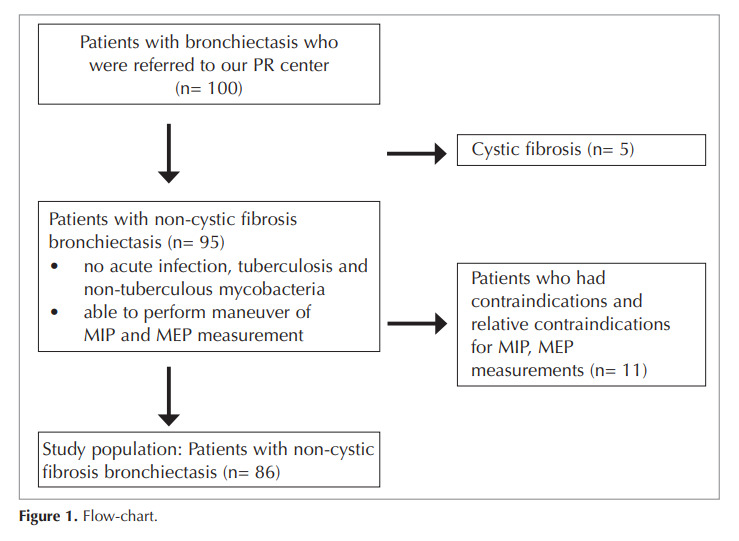
Consolidation with the appearance of a reversed halo sign in the lower lobe of the left lung.

Patients with NCFB referred to our hospital pulmonary rehabilitation center who had no acute infection (confirmed by history, serum C-reactive protein, chest X-ray, and HRCT) at the same time of evaluation, who were able to perform maneuvers of maximal inspiratory, expiratory pressure [maximum inspiratory pressure (MIP), maximum expiratory pressure (MEP)] measurements according to the recommendations of the American Thoracic Society and European Respiratory Society (ATS-ERS), and who had three negative sputum cultures of tuberculous and nontuberculous mycobacteria ( Figure 1 ). The exclusion criteria included contraindications and relative contraindications for MIP and MEP measurements, such as severe pulmonary hypertension, tympanic membrane problems, and large bullae observed in HRCT scans. All assessments, HRCT scans, and sputum culture analyses were performed at the same time. The number of hospitalizations and sputum cultures in a one-year period was investigated from hospital records and confirmed by the patients’ histories.

In line with the primary objective of the study, all patients were divided into two groups according to the presence of clinically important inspiratory respiratory muscle weakness according to guidelines, with a cut-off value of -80 cm H_2_O ( 10 , 11 ). The presence of any bacterial colonization and other parameters were compared between the two groups.

For the secondary objective of the study, all patients were divided into two groups according to the presence of any bacterial colonization, and MIP and MEP values and other parameters were compared between the groups.

### Outcomes Measures

Respiratory muscle strength was evaluated by measuring the MIP and MEP using a MicroRPM respiratory pressure meter (CareFusion, Hoechberg, Germany). MIP and MEP were measured with the subject in a sitting position by the same physiotherapist, in accordance with the recommendations of the ATS-ERS ( 10 ). MIP was measured starting from the residual volume, and MEP was measured starting from total lung capacity. Tests were repeated a minimum of three times, and the best value was recorded.

Colonization is defined as the presence of two or more consecutive cultures (or >50% of cultures) positive for the same potentially pathogenic microorganism within a period of at least six months in samples taken at least one month apart ( 12 , 13 ).

Exercise capacity was evaluated using the Incremental Shuttle Walking Test (ISWT) and Endurance Shuttle Walking Test (ESWT) ( 14 ). Health-related quality of life was assessed using the St. George’s Respiratory Questionnaire (SGRQ) ( 15 ) and chronic respiratory questionnaire (CRQ). Dyspnea was assessed using the Medical Research Council (MRC) scale ( 16 ).

Spirometry was performed to determine forced vital capacity (FVC), forced expiratory volume in one second (FEV_1_), and FEV_1_/FVC using a spirometer (AS507, Minato Medical Science, Tokyo, Japan), in accordance with the ATS-ERS guidelines 10. Bioelectrical impedance was used to assess body composition using a TANITA (TBF-300A Total Body Composition Analyzer, Tokyo, Japan). Body mass index (BMI) and fat-free mass index (FFMI) were calculated using the formula of weight (body mass for BMI, fat-free mass for FFMI) in kilograms divided by the square of the height in meters. Hospital Anxiety and Depression (HAD) scores were used to assess psychological status ( 17 ). The severity was assessed using the E-FACED score ( 18 ).

### Statistical Analysis

Statistical analyses were performed using the Statistical Package for the Social Sciences version 18.0 (SSPS, Chicago, IL, USA) software package. Statistical significance was determined as a probability value of <0.05. The minimum effect size (0.80) was calculated using Cohen’s d analysis in G-Power 3.1.9.2, considering an alpha level of 0.05 and a power (1-beta) of 0.80 (Heinrich-Heine-Universität, Düsseldorf, Germany) ( 19 ) according to the healthy population ( 20 ). Data are given as mean ± standard deviation and median (min-max). First, the variables were analyzed to assess the normality of distribution using the Shapiro-Wilk test. To assess relationships between categorical variables, the Chi-square test was used. The t-test or Mann-Whitney U test was used for the comparisons of groups. Linear regression (adjusting for age, sex, and BMI) was also used for comparing bacterial colonization groups in terms of MIP and MEP. Spearman correlation analyses were used for investigating relations between MIP and other variables.

## RESULTS

Forty-nine (57%) of 89 patients with NCFB were female. The mean age of the patients was 42 ± 13 years. Bronchiectasis etiologies were posttuberculosis (n= 10, 12%), post-infectious (n= 47, 55%), Kartagener’s syndrome (n= 7, 8%), primary ciliary dyskinesia (n= 2, 2%), and unknown (n= 20, 23%). Eighty (93%) patients had cystic bronchiectasis and six (7%) had varicose bronchiectasis. The median value of MIP was -68 (-16:-153) cm H_2_O, and MEP was 89 (16:256) cm H_2_O in all patients. The demographic features and parameters of all patients are given in Table 1. MIP values were correlated negatively with E-FACED scores (p= 0.005, r= -0.299) and SGRQ total scores (p= 0.033, r= -0.231); correlated positively with FEV_1_ (p= 0.024, r= 0.244) FVC-predicted % (p= 0.011, r= 0.284), and ISWT (p= 0.005, r= 0.301).

Fifty-eight (67%) patients had inspiratory muscle weakness (MIP < -80 cm H_2_O) with a median of 56 (16:80) cm H_2_O. The median value of MIP of those with no inspiratory muscle weakness was 96 (82:153) cm H_2_O. In the inspiratory muscle weakness group, 39 (67%) patients were female. The mean age was 43 ± 14 years. Forty-seven (81%) patients had never smoked. The mean value of MEP was 78 (16:144) cm H_2_O. The mean MRC score was 3 ± 1. The median value of FEV_1_-predicted was 41% (16:110). Twentyeight of 46 patients who had an obstruction in spirometry had inspiratory muscle weakness. As the median BMI value was 25 (15:40) kg/m2, this group was overweight. The median value of ISWT was 245 (40:580) m, and the mean ESWT was 8 (2:20) min. The mean value of anxiety score was 10 ± 2, the mean depression score was 10 ± 3, the mean SGRQ score was 61 ± 17, and the mean CRQ score was 71 ± 19 (Table 1). Twenty-two (40%) patients with inspiratory muscle weakness had severe E-FACED scores; 17 (29%) patients were moderate, and 19 (33%) were mild. In the other group, 18 (64%) patients were mild, six (21%) were moderate, and four (14%) were severe.

The inspiratory muscle weakness group had a higher number of females (p= 0.006), lower FEV_1_-predicted % (p= 0.044), FVC-predicted % (p= 0.021), ISWT (p= 0.002), CRQ scores (p= 0.012), and higher MRC scores (p< 0.001), E-FACED scores (p= 0.007), SGRQ scores (p= 0.008), and number of hospitalization in the previous year (p= 0.031). Although this group had lower FFMI, they were not statistically different from patients without respiratory muscle weakness. When patients with and without inspiratory muscle weakness were compared, the ratio of bacterial colonization, BMI, history of smoking, and HAD scores of each group was the same (Table 1 ).

**Table 1:** Demographic and clinical parameters of groups according to inspiratory muscle weakness

Parameters	MIP< -80 cm H_2_O n= 58	MIP≥ -80 cm H_2_O n= 28	Total	P*
Demographic Features				
Sex (m/f) n (%)	19 (33)	18 (64)	37 (43)	0.006
Sex (m/f) n (%)	39 (67)	10 (36)	49 (57)	0.006
Age (mean ± SD)/median (min-max)	43 ± 14	39 ± 4	42 ± 13	0.202
Age (mean ± SD)/median (min-max)	44 (18-70)	39 (20-59)	42 (18-82)	0.202
Smoking (never/current/ex-smoker) n (%)	47 (81)	19 (68)	68 (77)	0.394
Smoking (never/current/ex-smoker) n (%)	10 (72)	8 (29)	18 (21)	0.394
Smoking (never/current/ex-smoker) n (%)	1 (2)	1 (4)	2 (2)	0.394
Smoking (pack-year)	6 ± 18	5 ± 9	5 ± 15	0.134
Smoking (pack-year)	0 (0-90)	0 (0-30)	0 (0-90)	0.134
Clinical Outcomes				
MEP (cm H_2_O)	77 ± 26	130 ± 41	96 ± 41	<0.001
MEP (cm H_2_O)	78 (16:144)	127 (55:256)	89 (16:256)	<0.001
MRC score	3 ± 1	2 ± 1	3 ± 1	<0.001
MRC score	3 (1:5)	2 (1:4)	3 (1:5)	<0.001
FEV_1_%	43 ± 19	50 ± 18	45 ± 18	0.044
FEV_1_%	41 (16:110)	51 (23:85)	44 (16:110)	0.044
FVC%	52 ± 20	64 ± 23	56 ± 21	0.021
FVC%	49 (16:110)	69 (28:105)	53 (16:110)	0.021
FEV_1_/FVC	70 ± 12	68 ± 10	69 ± 11	0.518
BMI (kg/m2)	26 ± 7	25 ± 6	25 ± 6	0.880
BMI (kg/m2)	25 (15:40)	24 (15:38)	24 (15:40)	0.880
FFMI (kg/m2)	18 ± 7	19 ± 3	18 ± 3	0.052
FFMI (kg/m2)	17 (14:24)	18 (15:26)	18 (14:26)	0.052
ISWT (meters)	266 ± 140	373 ± 167	304 ± 157	0.002
ISWT (meters)	245 (40:580)	380 (40:630)	310 (40:630)	0.002
ESWT (min)	10 ± 7	11 ± 8	11 ± 7	0.292
ESWT (min)	8 (2:20)	11 (2:20)	9 (1.5:20)	0.292
SRGQ Total scores	61 ± 17	51 ± 19	57 ± 19	0.008
SRGQ Total scores	65 (26:98)	50 (21:88)	55 (21:98)	0.008
CRQ scores	71 ± 19	79 ± 19	74 ± 19	0.012
CRQ scores	71 (34:109)	79 (37:101)	75 (34:109)	0.012
HAD (Anxiety/depression) scores	10 ± 2 10 (5:14)	10 ± 2 10 (7:14)	10 ± 2 10	0.728
HAD (Anxiety/depression) scores	10 ± 3 10 (4:15)	9 ± 2 9 (5:13)	(5:14)	0.267
E-FACED scores	3 ± 2	2 ± 2	3 ± 2	0.007
E-FACED scores	4 (0:7)	1 (0:7)	3 (0:7)	0.007
Hospitalizations in the previous year (n)	1 ± 1	0 ± 1	1 ± 1	0.031
Hospitalizations in the previous year (n)	1 (0:2)	0 (0:2)	0 (0:2)	0.031
Affected lobes (n)	3 ± 1 3 (1:5)	3 ± 1 3 (1:5)	3 ± 1 3 (1:5)	0.131
Bacterial colonization(+) n(%)	16 (28)	8 (29)	24	0.924

*The t-test or Mann-Whitney U test was used for the comparisons of groups Variables were given as mean ± SD and median (min:max). MIP: Maximum inspiratory pressure, MEP: Maximum expiratory pressure, MRC: Medical Research Council, FVC: Forced vital capacity, FEV_1_: Forced expiratory volume in one second, BMI: Body mass index, FFMI: Fat- free mass index, ISWT: Incremental shuttle walking test, ESWT: Endurance shuttle walking test, SGRQ: St. George’s Respiratory Questionnaire, CRQ: Chronic respiratory questionnaire.

Twenty-four (28%) of 86 patients were colonized with bacteria. Twenty (83%) patients were colonized with Pseudomonas aeruginosa, two (7%) with Haemophilus influenzae, one (5%) with Streptococcus pneumoniae, and one (5%) with Moraxella catarrhalis. Most of the colonized patients were female. The mean age of this group was 43 ± 13 years. Eighteen (75%) colonized patients had never smoked. The median MIP and MEP were -73 (-20:-127) and 100 (40:173) cm H_2_O, respectively. The mean MRC score was 3 ± 1. The median value of FEV_1_-predicted was 33% (17:110). Thirteen patients of 46 patients who had an obstruction in spirometry had colonization. According to the median BMI value of 27 (15:40) kg/m2, this group was overweight. The median ISWT was 180 (40:540) m, and the median ESWT was 5 (1:20) min. The mean anxiety score of the colonized patients was 10 ± 1, and the mean depression score was 10 ± 2, both of which were borderline ( Table 2 ). The mean E-FACED severity score was 5 ± 2. Out of the colonized patients, thirteen (54%) were classified as severe, and seven (29%) were classified as moderate. Among the colonized patients, ten (42%) had not been hospitalized in the previous year, eight (33%) had been hospitalized once, and six (25%) had been hospitalized twice.

**Figure 2:**
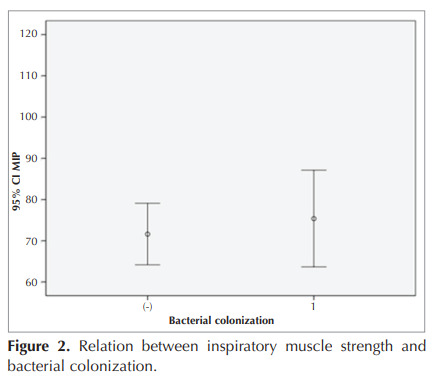
Consolidation with the appearance of a reversed halo sign in the lower lobe of the left lung.

When comparing the colonized and non-colonized patients, there were no statistically significant differences in MIP and MEP values, as shown in Figure 2. After adjusting for BMI, age, and sex, there were no significant differences in MIP (p= 0.442) and MEP (p= 0.639) values between the groups. The colonized patients had similar age, sex distribution, smoking history, HAD scores, BMI, and FFMI to the non-colonized patients. Although they had worse quality of life scores, they did not reach significance. FEV_1_ (p= 0.006), FVC (p= 0.031), ISWT (p= 0.034), and ESWT (p= 0.043) were lower, and E-FACED scores (p< 0.001) were higher in colonized patients ( Table 2).

## DISCUSSION

This study showed that patients with NCFB with inspiratory muscle weakness could have more severe disease, worse pulmonary function, quality of life, limited exercise capacity, and more hospitalizations, and could be more dyspneic than patients without inspiratory muscle weakness. Furthermore, in patients with NCFB, inspiratory muscle strength could be associated with pulmonary function, quality of life, exercise capacity, and disease severity. Also, it was shown that colonized patients could have worse quality of life and more hospitalizations, even though respiratory muscle weakness was found similar to non-colonized patients. In colonized patients, the most prominent difference was worse disease severity, pulmonary functions, and reduced exercise capacity.

In patients with bronchiectasis, major symptoms are cough, excessive secretions, dyspnea, exercise intolerance, and fatigue. The possible mechanisms for chronic colonization and exacerbations include unproductive cough, insufficient removal of secretions, and increased inflammation. Respiratory muscle weakness may contribute to these mechanisms. Respiratory muscle weakness in patients with bronchiectasis has been shown in a few studies ( 4 , 5 ). In one of these studies, the mean MIP value was -70, -74 cm H_2_O with 42% of FEV_1_- predicted in patients with NCFB. The values of MIP and FEV_1_ were similar to our study, but our patients were younger than in that study ( 4 ). In a study from Türkiye, even though the age, BMI, and distribution of sex were similar to our patients, respiratory muscle weakness was not shown and a better pulmonary function test and ISWT distance were revealed ( 21 ). This could be linked to the greater pack-year smoking history and worse pulmonary function tests of our patients. In several studies, the only two determinants of the six-minute walk test were found to be knee extension strength and MIP in patients with moderate and severe chronic obstructive pulmonary disease (COPD) ( 22 , 23 ). It was concluded that the presence of inspiratory muscle weakness might be associated with impaired pulmonary function in patients with COPD. In our study, the inspiratory muscle weakness group was more dyspneic and had worse pulmonary function, exercise capacity, and quality of life. MIP values were also found to correlate with pulmonary function, exercise capacity, and quality of life. These results suggest that the same mechanisms could be underlying in patients with COPD and NCFB. In patients with bronchiectasis, the combination of peripheral and respiratory muscle impairment results in exacerbations, fatigue, higher levels of anxiety, depression, and poorer quality of life ( 24 , 25 ). Similarly, in our study, patients with inspiratory muscle weakness had poorer body composition, higher severity scores, higher numbers of hospitalizations in the previous year, and worse quality of life, in addition to reduced exercise capacity.

**Table 2:** Demographic and clinical parameters of groups according to bacterial colonization

Parameters	Colonization (+) n= 24	Colonization (-) n= 62	p
Demographic Features			
Sex (m/f) n (%)	11 (46%)	26 (42%)	0.743
Sex (m/f) n (%)	13 (54%)	36 (58%)	0.743
Age (mean ± SD/median (min-max)	43 ± 13	41 ± 13	0.447
Age (mean ± SD/median (min-max)	43 (18-68)	40 (18-70)	0.447
Smoking (never/current/ex-smoker) n (%)	18 (75%)	48 (77%)	0.591
Smoking (never/current/ex-smoker) n (%)	6 (25%)	12 (20%)	0.591
Smoking (never/current/ex-smoker) n (%)	0 (0%)	2 (3%)	0.591
Smoking (pack-year)	2 ± 6	7 ± 18	0.863
Smoking (pack-year)	0 (0-30)	0 (0-90)	0.863
Clinical outcomes			
MIP (cm H_2_O)	77 ± 30	70 ± 28	0.433
MIP (cm H_2_O)	73 (20-127)	68 (16-153)	0.433
MEP (cm H_2_O)	100 ± 35	94 ± 43	0.700
MEP (cm H_2_O)	100 (40-173)	86 (16-256)	0.700
MRC score	3 ± 1	3 ± 1	0.402
MRC score	3 (1-5)	3 (1-5)	0.402
FEV_1_%	39 ± 22	48 ± 17	0.006
FEV_1_%	33 (17-110)	50 (16-84)	0.006
FVC%	48 ± 21	59 ± 21	0.031
FVC%	45 (19-110)	59 (16-105)	0.031
FEV_1_/FVC	69 ± 13	70 ± 10	0.865
FEV_1_/FVC	68 (46-92)	69 (45-98)	0.865
BMI (kg/m2)	26 ± 7	25 ± 6	0.650
BMI (kg/m2)	27 (15-40)	24 (15-39)	0.650
FFMI (kg/m2)	18 ± 3	18 ± 3	0.969
FFMI (kg/m2	18 (15-24)	18 (14-26)	0.969
ISWT (meters)	230 ± 147	331 ± 153	0.034
ISWT (meters)	180 (40-540)	345 (80-630)	0.034
ESWT (min)	7 ± 6	12 ± 7	0.043
ESWT (min)	5 (1-20)	12 (2-20)	0.043
SRGQ Total scores	61 ± 20	56 ± 19	0.175
SRGQ Total scores	64 (29-94)	55 (21-98)	0.175
CRQ scores	70 ± 19	75 ± 19	0.246
CRQ scores	70 (41-101)	76 (34-109)	0.246
HAD (Anxiety/depression) scores	10 ± 1 10 (7-13)	10 ± 2 10 (5-14)	0.385
HAD (Anxiety/depression) scores	10 ± 2 10 (5-14)	9 ± 2 9 (4-15)	0.481
E-FACED scores	5 ± 2	2 ± 2	<0.001
E-FACED scores	5 (1-7)	1 (0-6)	<0.001
Hospitalization in the previous year (n)	1 ± 1	1 ± 1	0.159
Hospitalization in the previous year (n)	1 (0-2)	0 (0-2)	0.159
Affected lobes (n)	3 ± 1	3 ± 1	0.031
Affected lobes (n)	3 (1-5)	3 (1-5)	0.031

*The t-test or Mann-Whitney U test was used for the comparisons of groups Variables were given as mean ± SD and median (min-max). MIP: Maximum inspiratory pressure, MEP: Maximum expiratory pressure, MRC: Medical research council, FVC: Forced vital capacity, FEV_1_: Forced expiratory volume in one second, BMI: Body mass index, FFMI: Fat-free mass index, ISWT: Incremental shuttle walking test, ESWT: Endurance shuttle walking test, SGRQ: St. George’s Respiratory Questionnaire, CRQ: Chronic respiratory questionnaire.

Bronchiectasis was a neglected disease, but interest in the condition has been increasing in recent times. The economic burden of bronchiectasis has been found to be similar to that of COPD, with costs decreasing as disease severity decreases. This includes reductions in hospitalizations, intensive care utilization, and the use of inhaled antibiotics ( 26 , 27 ). Bacterial colonization has been shown to increase the economic burden on individuals with non-cystic fibrosis bronchiectasis (NCFB) through increased inflammation, greater impairment of lung function, more exacerbations, higher mortality rates, and a deterioration of life quality ( 28 ). More than 50% of patients are chronically infected with bacteria ( 29 ). For long-term colonization, the most common agents are H. influenzae and P.s aeruginosa, S. pneumoniae, M. catarrhalis, S. aureus, and Burkholderia spp. are also widespread. In our study, 28% of patients were colonized with bacteria. Eighty-three percent of patients were colonized with P. aeruginosa, and other patients with H. influenzae, S. pneumoniae, and M. catarrhalis.

Bronchiectasis is usually diagnosed in the fifth or sixth decade of life, but the disease may be seen in all age groups ( 30 ). The rate of bacterial colonization was found to be similar when comparing the elderly cohort with patients aged <76 years in a previous study ( 31 ). In our study, the mean age of all patients was 42 ± 13 years, and the mean ages of colonized and non-colonized patients were similar. Although more than 50% of patients have airflow obstruction, lung function can vary widely in these patients. In a study, loss of FEV_1_ was found to be greater in colonized patients, and more severe bronchial dilatation resulted in increased airflow obstruction ( 32 ). Half of our patients had airflow obstruction. Similarly, the FEV_1_ values of colonized patients were significantly lower than those of non-colonized patients in our study. Besides pulmonary function tests, cross-sectional studies showed that patients with colonization had worse health-related quality of life per the SGRQ, and more frequent exacerbations ( 33 , 34 ), similar to our study. In a recent study, exercise capacity was decreased in patients with NCFB compared with healthy subjects ( 35 ). In our study, colonized patients had statistically lower ISWT [180 (40:540) min in the colonized group, 345 (80:630) m in the non-colonized group], and ESWT [5 (1:20) min, 12 (1:20) min, respectively]. Our patients had similar levels of dyspnea sensation, anxiety, and depression, regardless of colonization. These results could be due to the small number of patients or the fact that all patients who were referred to our PR center because of these symptoms and findings, had already been symptomatic and had a worse health-related quality of life, and physiological status.

BMI has been identified as another important clinical and prognostic factor in patients with non-cystic fibrosis bronchiectasis (NCFB), as indicated by several studies. It was shown that as BMI increase, the rate of colonization decreases ( 36 ). In our study, it was observed that all patients, regardless of colonization, were overweight. Furthermore, after adjusting for BMI, age, and sex, similar respiratory muscle strengths were found between the colonized and non-colonized groups. Moreover, the ratio of bacterial colonization was found to be similar irrespective of the presence of inspiratory muscle weakness. These findings suggest that there is no direct association between bacterial colonization and inspiratory muscle weakness.

The main limitation of this study was its nonrandomized design. While the study captured reallife experiences and data from a pulmonary rehabilitation center in a referral center in the capital city of Türkiye, it was limited to a single center. Therefore, the findings may not be applicable to a broader population. Another significant limitation of the study was the lack of information on vaccination history and disease duration.

## CONCLUSION

While there was no direct association between inspiratory muscle strength and bacterial colonization in patients with NCFB, it is worth noting that inspiratory muscle weakness can have implications for disease severity, pulmonary function, quality of life, and exercise capacity. Bacterial colonization, on the other hand, was found to be associated with increased disease severity, worsened pulmonary function, and reduced exercise capacity. This study highlights the significance of examining both inspiratory muscle weakness and colonization in order to better understand the condition.

## Conflict of INTEREST

This study was approved by the Ankara Atatürk Chest Diseases and Thoracic Surgery Training and Research Hospital Ethics Committee (Decision no: 646, Decision date: 17.10.2019).

## Conflict of INTEREST

The authors declare that they have no conflict of interest

## AUTHORSHIP CONTRIBUTIONS

Concept/Design: İC

Analysis/Interpretation: İC, PE

Data acqusition: İC, ND, FT

Writing: All of authors

Clinical Revision: All of authors

Final Approval: All of authors
